# Hypomagnesemia and increased risk of new-onset diabetes mellitus after transplantation in pediatric renal transplant recipients

**DOI:** 10.1007/s00467-016-3571-6

**Published:** 2016-12-30

**Authors:** Wesley Hayes, Sheila Boyle, Adrian Carroll, Detlef Bockenhauer, Stephen D. Marks

**Affiliations:** 1 Great Ormond Street Hospital for Children, London, WC1N 3JH UK; 2 University College London Institute of Child Health, London, UK

**Keywords:** Kidney transplantation, Diabetes, Hypomagnesemia, Children, Tacrolimus

## Abstract

**Background:**

New-onset diabetes after transplantation (NODAT) is a significant co-morbidity following kidney transplantation. Lower post-transplant serum magnesium levels have been found to be an independent risk factor for NODAT in adult kidney transplant recipients.

**Methods:**

We undertook a retrospective analysis of risk factors for NODAT in pediatric renal transplant recipients at our institution with the aim of determining if hypomagnesemia confers a significant risk of developing NODAT in this patient population.

**Results:**

A total of 173 children with a median age at transplantation of 7.0 (range 1.3–17.5) years were included. Hypomagnesemia was found to be a significant independent risk factor for NODAT (*p* = 0.01). High trough tacrolimus levels were also independently associated with NODAT (*p* < 0.001). There was no significant association between NODAT and body mass index at the time of transplantation, monthly cumulative steroid dose or post-transplant cytomegalovirus viremia (*p* = 0.9, 0.6 and 0.7, respectively).

**Conclusions:**

This study identifies hypomagnesemia as a significant independent risk factor for the development of NODAT in pediatric renal transplant recipients. Given the clear association between hypomagnesemia and NODAT in both adults and children following renal transplantation, further studies are merited to clarify the etiology of this association and to examine the effect of magnesium supplementation on NODAT.

## Introduction

New-onset diabetes after transplantation (NODAT) is a significant co-morbidity in kidney transplant recipients. It compromises both renal transplant and patient survival [1]. The incidence of NODAT is increasing in pediatric renal transplant recipients, although it remains less prevalent than in the adult population [2]. NODAT adds to the burden of cardiovascular disease observed in children following kidney transplantation [3, 4]. A deeper understanding of this co-morbid disease is needed in order to inform strategies to aid its prevention and improve clinical management.

In adult kidney transplant recipients, a number of independent risk factors for NODAT have been identified, including obesity, family history of diabetes, ethnicity, older age, infections and episodes of acute rejection [5–9]. Known risk factors for NODAT in pediatric kidney transplant recipients include increasing age, body mass index (BMI) of >30 kg/m^2^, tacrolimus exposure and primary cytomegalovirus (CMV) mismatch [2].

Lower post-transplant serum magnesium levels have been found to be an independent risk factor for NODAT in adult kidney transplant recipients [10]. Whilst hypomagnesemia is prevalent in children following renal transplantation [11], its association with NODAT has not previously been examined.

We undertook a retrospective analysis of risk factors for NODAT in 173 pediatric renal transplant recipients at our institution with the aim of determining if hypomagnesemia confers a significant risk of developing NODAT in children.

## Methods

A retrospective analysis of a clinical database of pediatric renal transplant recipients transplanted and followed at our institution was undertaken. Patients transplanted after 1 January 2000 with a minimum 3 months of follow-up data, including laboratory test results, were eligible. Exclusion criteria were insulin therapy prior to transplant for diabetes mellitus or metabolic disease. Data were pseudo-anonymized for analysis. Local research ethics approval was obtained.

A NODAT episode was defined as two or more consecutive random blood glucose measurements of >11.1 mmol/l taken on separate days, excluding the first 7 days post-transplantation. CMV viremia was defined by positive detection of CMV PCR DNA in whole blood. A 30-day moving average trough tacrolimus level of >12 ng/l was defined as high.

The cumulative probability of NODAT over time was expressed using survival analysis. Cox proportional hazard models were fitted to determine the significance of various risk factors for NODAT. Covariates included patient age, BMI, plasma magnesium levels, trough tacrolimus levels, recipient CMV viremia post-transplant, cumulative monthly dose of prednisolone and mutations in the hepatocyte nuclear factor-1beta (*HNF-1B*) gene known to be causative of renal cysts and diabetes (RCAD) syndrome.

Monthly (30 day) moving average values of continuous covariates commencing day 30 post-transplantation were used to determine their effect on NODAT risk as time progresses. Colinearity between continuous covariates was analyzed using Pearson’s correlation.

All analyses were performed using Stata IC, version 13 (StataCorp LP, College Station, TX).

## Results

A review of the medical files resulted in the identification of 175 children transplanted between 1 January 2000 and 31 May 2016 and currently followed at our center with sufficient data for analysis and therefore eligible for entry to our study. Two patients were subsequently excluded as they were receiving insulin therapy prior to transplantation. The median age at transplantation was 7.0 (range 1.3–17.5) years, and 101 (58%) of the children were male. The median follow-up was 4.2 (range 0.6–15.9) years.

Of the 173 pediatric renal transplant recipients enrolled in our study, 20 (11%) experienced NODAT, with eight (5%) children requiring insulin therapy for >12 months. The median time to detection of NODAT was 9 (range 9–551) days. Three patients developed NODAT between 8 and 30 days post-transplantation, one of whom required insulin therapy for >12 months.

Five (3%) of the 173 children had sustained hypomagnesemia on the 30-day moving average assessments. The median BMI at the time of transplantation was 17.9 (range 13.8–33.6) kg/m^2^. Median monthly moving average trough tacrolimus levels were 3.9 (range 2.0–15.4) ng/l. Forty one (24%) children had high monthly moving average trough tacrolimus levels, and 49 (31%) children had CMV viremia post-transplantation during the study period. The median cumulative prednisolone dose was 793 (range 720–3430) mg/m^2^ body surface area for the first 30 days post-transplantation. Ten (6%) children with ultrasound findings consistent with RCAD were offered genetic testing of* HNF-1B*, and a causative* HNF-1B* gene deletion was found in one child.

**Fig. 1:**
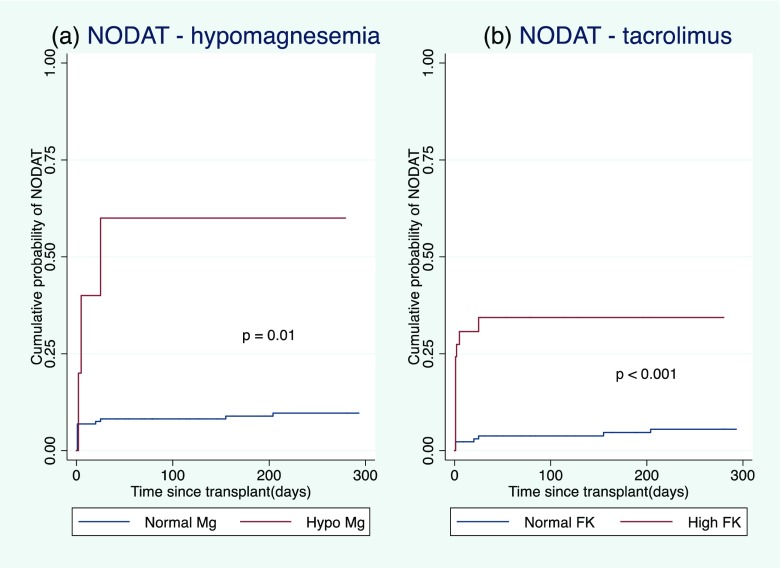
Cumulative incidence of new-onset diabetes after transplantation (*NODAT*) in children with normal and low plasma magnesium (*Mg*) levels (**a**) and normal and high trough tacrolimus levels (**b**). *FK* tacrolimus

Hypomagnesemia was found to be a significant independent risk factor for developing NODAT (*p* = 0.01;Fig. 1a). High trough tacrolimus levels were also independently associated with NODAT (*p* < 0.001; Fig. 1b). No significant association between NODAT and post-transplant CMV viremia was observed (*p* = 0.7; Table 1). Similarly, children’s BMI at the time of transplantation was not significantly associated with NODAT (*p* = 0.9; Table 1). No association between cumulative 30-day prednisolone dose normalized to body surface area and NODAT was observed (*p* = 0.6; Table 1). Insufficient genetic data were available to reliably determine the association between* HNF-1B* mutations and NODAT.

**Table 1:** Risk factors for new-onset diabetes after transplantation as determined by Cox proportional hazards model

Risk factor	Hazard ratio	95% Confidence interval	*p* value
High 30-day moving average trough tacrolimus level	5.9	2.2–15.3	<0.001
Low 30-day moving average magnesium level	4.6	1.4–14.6	0.01
Body mass index at transplantation	1.0	0.9–1.1	0.9
Cytomegalovirus viremia post transplantation	0.8	0.3–2.2	0.7
Age at transplantation	0.9	0.8–1.1	0.8
Cumulative 30-day prednisolone dose	0.9	1.0	0.6

*NODAT* new onset diabetes after transplantation

**Fig. 2:**
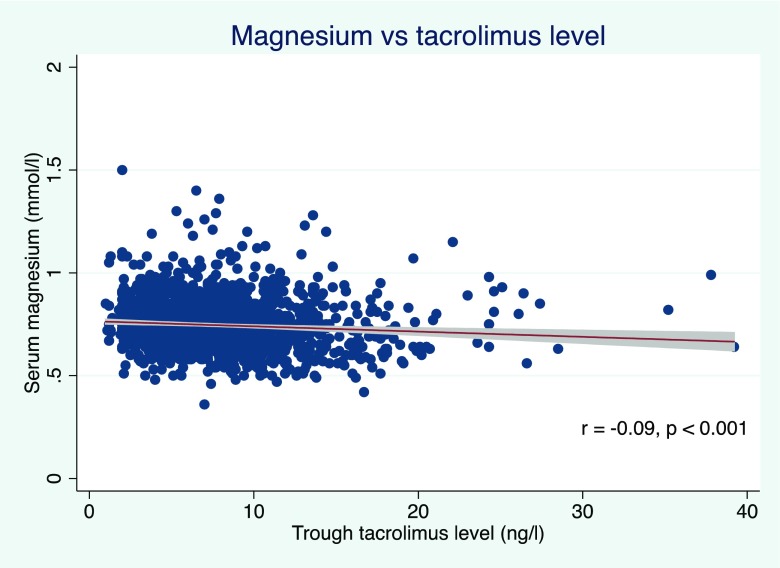
Linear relationship between serum magnesium and trough tacrolimus levels. (Best fit linear regression line with 95% confidence intervals shown)

The association between hypomagnesemia and tacrolimus levels was examined in order to determine if these risk factors for NODAT were independent. Of the 41 children with high trough tacrolimus levels, three (7%) had hypomagnesemia. No significant association was found between patients with hypomagnesemia and high trough tacrolimus levels (analysis of variance *p* = 0.06). A small negative linear correlation between serum magnesium levels and trough tacrolimus levels was observed (*r* = −0.09, *p* < 0.001; Fig. 2).

## Discussion

This study identifies hypomagnesemia as a significant independent risk factor for NODAT in pediatric renal transplant recipients. High trough tacrolimus levels confer additional risk, which was found to be independent of hypomagnesemia. Neither BMI, patient age or CMV viremia were significantly associated with NODAT in this cohort.

Several risk factors for NODAT in pediatric renal transplant recipients have been reported in previous studies. These include increasing age, BMI, tacrolimus exposure and primary CMV mismatch [2]. Some of these risk factors were corroborated in the current study, whilst others were not replicated in our data. The association of calcineurin inhibition with NODAT was clearly seen in the present study. The cumulative effect of high tacrolimus levels significantly increased the risk of NODAT in this pediatric cohort. Other previously reported NODAT risk factors were not replicated in the present analysis, namely age at transplantation, BMI and CMV viremia. This discordance may be related to limitations of the present study, such as its retrospective design and relatively small numbers, both of which may compromise detection of a true association between risk factors and the development of NODAT. Alternatively, the lack of reproducibility of some NODAT risk factors in the current data may simply reflect the absence of a robust association in children.

The effect of transplant immunosuppression regimens on the risk of NODAT remains debated. Randomized trials of early corticosteroid withdrawal in adult renal transplant recipients showed limited impact on NODAT [12, 13], whereas lower rates of hyperglycemia and NODAT were reported in pediatric renal transplant recipients following early corticosteroid withdrawal [14, 15]. The current study showed no significant association between monthly cumulative steroid exposure and NODAT. The lack of an observed relationship may relate to predominance of other NODAT risk factors, such as tacrolimus exposure or hypomagnesemia. Alternatively, a true association between corticosteroid exposure and NODAT may be confounded by the limitations of the current study outlined below.

To the authors’ knowledge, this is the first report of the association between hypomagnesemia and NODAT in pediatric renal transplant recipients. A clear link with NODAT has been established in adult kidney transplant recipients [10, 16–18]. The pathophysiological mechanism underlying the association between hypomagnesemia and NODAT has not yet been elucidated. Several observations suggest that a causal relationship is biologically plausible. Over two decades ago, magnesium was found to be an important cofactor in pancreatic insulin secretion and cellular glucose uptake [19, 20]. Magnesium deficiency has been shown to promote insulin resistance [21–23]. Supplementation of magnesium is reported to improve glucose tolerance in animal and clinical studies in non-transplant patients with diabetes mellitus [24–30]. A definitive causal link between hypomagnesemia and NODAT remains to be found.

Alternatively, the association between hypomagnesemia and NODAT may not be causal. Both hypomagnesemia and diabetes can result from mitochondrial dysfunction, as found in a large pedigree with hypomagnesemia and metabolic syndrome [31]. Mitochondrial dysfunction could therefore underlie the association between hypomagnesemia and NODAT. In kidney transplant recipients, tacrolimus can cause secondary mitochondrial respiratory chain dysfunction [32–34]. This could therefore be the common link in the association between hypomagnesemia and NODAT. Further work is needed to definitively establish whether the link between hypomagnesemia and NODAT in pediatric renal transplant recipients is purely an association, or a causal relationship.

Given the significant association of both hypomagnesemia and high trough tacrolimus levels with NODAT in the present study, we examined the association between these two covariates. Hypomagnesemia is reported as occurring in 3–48% of patients taking tacrolimus [35–37]. Calcineurin inhibition has been implicated in hypomagnesemia via downregulation of renal tubular calcium and magnesium reabsorption in several previous studies [38–41]. We observed no significant association between hypomagnesemia and high trough tacrolimus levels, while a direct relationship between plasma magnesium and trough tacrolimus levels was evident, albeit with a relatively “flat” gradient. These observations, taken together with the Cox proportional hazards model, suggest that hypomagnesemia is an independent risk factor for NODAT, with tacrolimus being a separate independent risk factor.

There are several motivations to urgently find effective interventions to reduce the risk of developing NODAT in children. NODAT presents particular challenges in the pediatric population. Glycemic control is challenging to achieve in growing children and adolescents [42, 43]. Adolescence itself is a potent risk factor for renal transplant loss, probably due to variable adherence to immunosuppressive medication [44]. Concordance with prescribed insulin and blood glucose monitoring in addition to transplant immunosuppression and fluid regimens is difficult for even the most motivated families. The burden of cardiovascular disease in pediatric kidney transplant recipients is substantial, with significant additional cumulative cardiovascular risk from NODAT in affected children. Effective interventions to reduce this risk are needed.

Hypomagnesemia is a potentially modifiable risk factor for NODAT. Supplementation of magnesium in adult kidney transplant recipients was found to improve fasting blood glucose levels 3 months post-transplant in a randomized study [45]. The effect of magnesium supplementation on the risk of NODAT remains unclear as this study was not powered to evaluate this. Given the well-established relationship of NODAT with hypomagnesemia, a well-powered study to evaluate the effect of magnesium supplementation on NODAT risk in adult and pediatric kidney transplant recipients is merited.

This study has a number of limitations. Firstly, its retrospective design limits the completeness of data and the ability to apply rigorous entry criteria. This weakness was mitigated in part by using a large volume of electronic test results for analyses exported from a clinical laboratory system. Secondly, relative to studies on adult kidney transplant recipients, the number of participants is small at 173, which limits the impact of the findings somewhat. The results obtained were nevertheless clinically significant. Thirdly, genetic testing was not performed on all patients, which limited the assessment of the contribution* HNF-1B* mutations to both hypomagnesemia and NODAT. Notwithstanding these limitations, these data clearly demonstrate a significant association between hypomagnesemia and NODAT in children.

This study identifies a clear association between hypomagnesemia and NODAT in children following kidney transplantation. Further work is needed to determine the exact etiology of this association, in particular the genetic contribution from candidate genes such as* HNF-1B*. Given the evidence of the link between NODAT and hypomagnesemia in both adult and children following renal transplantation, interventional studies to understand the effect of magnesium supplementation on NODAT risk are merited.

## References

[1] Cosio FG, Pesavento TE, Kim S, Osei K, Henry M, Ferguson RM (2002). Patient survival after renal transplantation: IV. Impact of post-transplant diabetes. Kidney Int.

[2] Burroughs TE, Swindle JP, Salvalaggio PR, Lentine KL, Takemoto SK, Bunnapradist S, Brennan DC, Schnitzler MA (2009). Increasing incidence of new-onset diabetes after transplant among pediatric renal transplant patients. Transplantation.

[3] Silverstein DM (2004). Risk factors for cardiovascular disease in pediatric renal transplant recipients. Pediatr Transplant.

[4] Becker-Cohen R, Nir A, Rinat C, Feinstein S, Algur N, Farber B, Frishberg Y (2006). Risk factors for cardiovascular disease in children and young adults after renal transplantation. Clin J Am Soc Nephrol.

[5] Bodziak KA, Hricik DE (2009). New-onset diabetes mellitus after solid organ transplantation. Transpl Int.

[6] Davidson JA, Wilkinson A, International Expert Panel on New-Onset Diabetes after Transplantation (2004). New-onset diabetes after transplantation 2003 international consensus guidelines: an endocrinologist’s view. Diabetes Care.

[7] Kaposztas Z, Gyurus E, Kahan BD (2011). New-onset diabetes after renal transplantation: diagnosis, incidence, risk factors, impact on outcomes, and novel implications. Transplant Proc.

[8] Kasiske BL, Snyder JJ, Gilbertson D, Matas AJ (2003). Diabetes mellitus after kidney transplantation in the United States. Am J Transplant.

[9] Hjelmesaeth J, Sagedal S, Hartmann A, Rollag H, Egeland T, Hagen M, Nordal KP, Jenssen T (2004). Asymptomatic cytomegalovirus infection is associated with increased risk of new-onset diabetes mellitus and impaired insulin release after renal transplantation. Diabetologia.

[10] Huang JW, Famure O, Li Y, Kim SJ (2016). Hypomagnesemia and the risk of new-onset diabetes mellitus after kidney transplantation. J Am Soc Nephrol.

[11] Uslu Gokceoglu A, Comak E, Dogan CS, Koyun M, Akbas H, Akman S (2014). Magnesium excretion and hypomagnesemia in pediatric renal transplant recipients. Ren Fail.

[12] Pirsch JD, Henning AK, First MR, Fitzsimmons W, Gaber AO, Reisfield R, Shihab F, Woodle ES (2015). New-onset diabetes after transplantation: results from a double-blind early corticosteroid withdrawal trial. Am J Transplant.

[13] Woodle ES, First MR, Pirsch J, Shihab F, Gaber AO, Van Veldhuisen P, Astellas Corticosteroid Withdrawal Study G (2008). A prospective, randomized, double-blind, placebo-controlled multicenter trial comparing early (7 days) corticosteroid cessation versus long-term, low-dose corticosteroid therapy. Ann Surg.

[14] Grenda R, Watson A, Trompeter R, Tonshoff B, Jaray J, Fitzpatrick M, Murer L, Vondrak K, Maxwell H, van Damme-Lombaerts R, Loirat C, Mor E, Cochat P, Milford DV, Brown M, Webb NJ (2010). A randomized trial to assess the impact of early steroid withdrawal on growth in pediatric renal transplantation: the TWIST study. Am J Transplant.

[15] Li L, Chang A, Naesens M, Kambham N, Waskerwitz J, Martin J, Wong C, Alexander S, Grimm P, Concepcion W, Salvatierra O, Sarwal MM (2009). Steroid-free immunosuppression since 1999: 129 pediatric renal transplants with sustained graft and patient benefits. Am J Transplant.

[16] Cheungpasitporn W, Thongprayoon C, Harindhanavudhi T, Edmonds PJ, Erickson SB (2016). Hypomagnesemia linked to new-onset diabetes mellitus after kidney transplantation: a systematic review and meta-analysis. Endocr Res.

[17] Sinangil A, Celik V, Barlas S, Sakaci T, Koc Y, Basturk T, Akin EB, Ecder T (2016). New-onset diabetes after kidney transplantation and pretransplant hypomagnesemia. Prog Transplant.

[18] Van Laecke S, Van Biesen W, Verbeke F, De Bacquer D, Peeters P, Vanholder R (2009). Posttransplantation hypomagnesemia and its relation with immunosuppression as predictors of new-onset diabetes after transplantation. Am J Transplant.

[19] Grafton G, Baxter MA (1992). The role of magnesium in diabetes mellitus. A possible mechanism for the development of diabetic complications. J Diabetes Complicat.

[20] Tosiello L (1996). Hypomagnesemia and diabetes mellitus. A review of clinical implications. Arch Intern Med.

[21] Yajnik CS, Smith RF, Hockaday TD, Ward NI (1984). Fasting plasma magnesium concentrations and glucose disposal in diabetes. Br Med J (Clin Res Ed).

[22] Alzaid AA, Dinneen SF, Moyer TP, Rizza RA (1995). Effects of insulin on plasma magnesium in noninsulin-dependent diabetes mellitus: evidence for insulin resistance. J Clin Endocrinol Metab.

[23] Nadler JL, Buchanan T, Natarajan R, Antonipillai I, Bergman R, Rude R (1993). Magnesium deficiency produces insulin resistance and increased thromboxane synthesis. Hypertension.

[24] Balon TW, Jasman A, Scott S, Meehan WP, Rude RK, Nadler JL (1994). Dietary magnesium prevents fructose-induced insulin insensitivity in rats. Hypertension.

[25] Sjogren A, Floren CH, Nilsson A (1988). Oral administration of magnesium hydroxide to subjects with insulin-dependent diabetes mellitus: effects on magnesium and potassium levels and on insulin requirements. Magnesium.

[26] Paolisso G, Sgambato S, Pizza G, Passariello N, Varricchio M, D’Onofrio F (1989). Improved insulin response and action by chronic magnesium administration in aged NIDDM subjects. Diabetes Care.

[27] Paolisso G, Passariello N, Pizza G, Marrazzo G, Giunta R, Sgambato S, Varricchio M, D’Onofrio F (1989). Dietary magnesium supplements improve B-cell response to glucose and arginine in elderly non-insulin dependent diabetic subjects. Acta Endocrinol (Copenh).

[28] Lopez-Ridaura R, Willett WC, Rimm EB, Liu S, Stampfer MJ, Manson JE, Hu FB (2004). Magnesium intake and risk of type 2 diabetes in men and women. Diabetes Care.

[29] Song Y, Manson JE, Buring JE, Liu S (2004). Dietary magnesium intake in relation to plasma insulin levels and risk of type 2 diabetes in women. Diabetes Care.

[30] Villegas R, Gao YT, Dai Q, Yang G, Cai H, Li H, Zheng W, Shu XO (2009). Dietary calcium and magnesium intakes and the risk of type 2 diabetes: the Shanghai Women’s Health Study. Am J Clin Nutr.

[31] Wilson FH, Hariri A, Farhi A, Zhao H, Petersen KF, Toka HR, Nelson-Williams C, Raja KM, Kashgarian M, Shulman GI, Scheinman SJ, Lifton RP (2004). A cluster of metabolic defects caused by mutation in a mitochondrial tRNA. Science.

[32] Tavira B, Gomez J, Diaz-Corte C, Llobet L, Ruiz-Pesini E, Ortega F, Coto E (2014). Mitochondrial DNA haplogroups and risk of new-onset diabetes among tacrolimus-treated renal transplanted patients. Gene.

[33] Palacin M, Coto E, Llobet L, Pacheu-Grau D, Montoya J, Ruiz-Pesini E (2013). FK506 affects mitochondrial protein synthesis and oxygen consumption in human cells. Cell Biol Toxicol.

[34] Simon N, Morin C, Urien S, Tillement JP, Bruguerolle B (2003). Tacrolimus and sirolimus decrease oxidative phosphorylation of isolated rat kidney mitochondria. Br J Pharmacol.

[35] Peters DH, Fitton A, Plosker GL, Faulds D (1993). Tacrolimus. A review of its pharmacology, and therapeutic potential in hepatic and renal transplantation. Drugs.

[36] Fung JJ, Starzl TE (1995). FK506 in solid organ transplantation. Ther Drug Monit.

[37] Shapiro R, Jordan ML, Scantlebury VP, Vivas C, Fung JJ, McCauley J, Randhawa P, Demetris AJ, Irish W, Mitchell S, Hakala TR, Starzl TESRL (1995). A prospective randomized trial of FK506-based immunosuppression after renal transplantation. Transplantation.

[38] Markell MS, Altura BT, Sarn Y, Barbour R, Friedman EA, Altura BM (1993). Relationship of ionized magnesium and cyclosporine level in renal transplant recipients. Ann N Y Acad Sci.

[39] Mazzola BL, Vannini SD, Truttmann AC, von Vigier RO, Wermuth B, Ferrari P, Bianchetti MG (2003). Long-term calcineurin inhibition and magnesium balance after renal transplantation. Transpl Int.

[40] Navaneethan SD, Sankarasubbaiyan S, Gross MD, Jeevanantham V, Monk RD (2006). Tacrolimus-associated hypomagnesemia in renal transplant recipients. Transplant Proc.

[41] Nijenhuis T, Hoenderop JG, Bindels RJ (2004). Downregulation of Ca(2+) and Mg(2+) transport proteins in the kidney explains tacrolimus (FK506)-induced hypercalciuria and hypomagnesemia. J Am Soc Nephrol.

[42] Drash AL (1993). The child, the adolescent, and the diabetes control and complications trial. Diabetes Care.

[43] Vollrath ME, Landolt MA, Gnehm HE, Laimbacher J, Sennhauser FH (2007). Child and parental personality are associated with glycaemic control in Type 1 diabetes. Diabet Med.

[44] Van Arendonk KJ, James NT, Boyarsky BJ, Garonzik-Wang JM, Orandi BJ, Magee JC, Smith JM, Colombani PM, Segev DL (2013). Age at graft loss after pediatric kidney transplantation: exploring the high-risk age window. Clin J Am Soc Nephrol.

[45] Van Laecke S, Nagler EV, Taes Y, Van Biesen W, Peeters P, Vanholder R (2014). The effect of magnesium supplements on early post-transplantation glucose metabolism: a randomized controlled trial. Transpl Int.

